# Echocardiography Combined With Radiomics and Deep Transfer Learning to Diagnose Hypertrophic Cardiomyopathy and Other Etiologies of Left Ventricular Hypertrophy: A Multicenter Study Comparing the Performance With Echocardiologists

**DOI:** 10.31083/RCM42800

**Published:** 2025-11-26

**Authors:** Jiangtao Wang, Sensen Wang, Tao Yu, Wensheng Tao, Haixin Shao, Caiyun Xia, Biaohu Liu

**Affiliations:** ^1^Department of Ultrasound Medicine, The First Affiliated Hospital of Wannan Medical College, 241001 Wuhu, Anhui, China; ^2^School of Health Science and Engineering, Ma'anshan University, 243100 Maanshan, Anhui, China; ^3^Department of Radiology, The First Affiliated Hospital of Chongqing Medical University, 400016 Chongqing, China; ^4^Department of Ultrasound Medicine, Zhejiang People's Hospital, 310003 Hangzhou, Zhejiang, China; ^5^Department of Ultrasound Medicine, Wuhu Second People's Hospital, 241100 Wuhu, Anhui, China

**Keywords:** radiomics, deep transfer learning, transthoracic echocardiography, hypertrophic cardiomyopathy, left ventricular hypertrophy

## Abstract

**Background:**

Hypertrophic cardiomyopathy (HCM) and left ventricular hypertrophy (LVH) from other causes present similar features on transthoracic echocardiography (TTE), making an accurate differentiation challenging. Recent advancements in radiomics and deep transfer learning (DTL) have shown promise; however, no studies have combined these techniques to diagnose HCM and LVH resulting from other causes. Therefore, we developed a fusion model that integrates radiomic features from the left ventricular myocardium in the four-chamber view of TTE with DTL features to differentiate HCM from other causes of LVH, providing more reliable diagnostic support.

**Methods:**

This multicenter study included 971 patients (303 with HCM, 668 with hypertensive heart disease and uremic cardiomyopathy). Patients from Institution 1 were split into a training set and an internal validation set, while patients from Institution 2 served as an external validation set. Radiomic features were extracted using pyradiomics, and DTL features were obtained via DenseNet121. Features were selected using least absolute shrinkage and selection operator (LASSO) and input into ten machine learning algorithms, with support vector machine (SVM) as the classifier. Model performance was assessed using receiver operating characteristic (ROC) curves and decision curve analysis (DCA) and compared with the diagnostic results of two ultrasound physicians.

**Results:**

The fusion model demonstrated excellent diagnostic performance: the area under the curve (AUC) values were 0.966 (training set), 0.945 (internal validation), and 0.934 (external validation), thereby outperforming models that used only radiomic or DTL features. DCA indicated superior clinical effectiveness, surpassing the diagnostic performance of two ultrasound physicians.

**Conclusions:**

A fusion model combining radiomics and DTL features significantly improves the ability to distinguish HCM from other causes of LVH and has strong potential for clinical applications.

## 1. Introduction 

Hypertrophic cardiomyopathy (HCM) is a genetic disease caused by gene mutations, with the primary feature of left ventricular hypertrophy (LVH) [1]. Additionally, hypertensive heart disease (HHD) and uremic cardiomyopathy (UCM) are also common causes of LVH [2, 3]. These three diseases differ significantly in terms of treatment and clinical management: patients with HCM require symptom control and prevention of sudden cardiac death; patients with HHD require blood pressure control; and UCM patients need dialysis treatment [1, 4, 5]. However, all three diseases present with LVH and exhibit similar findings on transthoracic echocardiography (TTE), making it difficult for echocardiographers to differentiate between HCM and other causes of LVH based solely on TTE. Therefore, developing a diagnostic method that is both accurate and reliable to effectively distinguish HCM from other causes of LVH is crucial for formulating personalized treatment plans and assessing patient prognosis.

The diagnosis of cardiovascular diseases typically relies on imaging examinations, including cardiac magnetic resonance imaging (MRI) and TTE [6, 7]. Compared to cardiac MRI, TTE, as an imaging technique, offers advantages such as ease of operation, low cost, and repeatability, making it widely used in the diagnosis of cardiovascular diseases [7]. However, the current analysis of TTE mainly depends on the clinical experience and qualitative judgment of echocardiographers and lacks quantitative analysis methods for the complex features of TTE. Therefore, extracting high-dimensional features with diagnostic significance from TTE images remains a key challenge that needs to be addressed.

In recent years, the integration of radiomics and deep learning (DL) technologies has opened new research prospects for medical image analysis [8, 9, 10]. Radiomics extract rich high-dimensional features from medical images, including texture, shape, and intensity, providing valuable information for the early diagnosis of diseases [11, 12, 13]. Meanwhile, DL, especially convolutional neural network (CNN), demonstrates tremendous potential in image recognition and analysis by extracting image features through filter matrices [14, 15]. However, in medical imaging applications, these methods often rely on large training datasets, which are typically difficult to obtain. Deep transfer learning (DTL) expands the application range of DL by automatically learning meaningful features from vast image data. With DTL, pre-trained models can quickly adapt to new tasks and smaller datasets, achieving remarkable results in various medical image analysis domains [16, 17, 18]. DTL not only extracts complex patterns but also surpasses traditional handcrafted features, significantly improving diagnostic accuracy. In recent years, the combination of quantitative features in radiomics and the powerful image processing capabilities of DTL has become a research hotspot. The synergistic effects of both have led to outstanding research outcomes in multiple areas, including the differentiation of benign and malignant breast nodules, prediction of lung nodule metastasis, identification of cervical lymph node metastasis in oral squamous cell carcinoma, classification of COVID-19 and non-COVID-19 pneumonia, preoperative staging of laryngeal cancer, and detection of occult peritoneal metastasis in pancreatic ductal adenocarcinoma patients [9, 19, 20, 21, 22, 23].

Differentiating HCM from LVH caused by other factors typically relies on complex diagnostic methods such as cardiac MRI, endomyocardial biopsy (EMB), and genetic Validation. These diagnostic procedures not only impose a significant financial burden on patients, but they are also time-consuming, labor-intensive, and in some cases, may still fail to provide a definitive diagnosis [24]. Previous studies have primarily focused on extracting single radiomic or DL features from cardiac MRI images to differentiate HCM from HHD [25, 26, 27]. Additionally, some studies have utilized DL features extracted from TTE to diagnose the etiology of LVH [28, 29, 30]. However, no study has yet combined radiomic features with DTL features for the differentiation of HCM from LVH caused by other factors. Building upon this, the current study proposes an innovative model that integrates radiomic and DTL features, specifically designed for the analysis of LVH regions in ultrasound images. This model provides a novel diagnostic approach for distinguishing HCM from other types of LVH. The results indicate that the fusion model (combines radiomics features and DTL features) excels in differentiating HCM from LVH of other etiologies, offering clinicians more precise diagnostic support.

## 2. Materials and Methods

**Fig. 1.**
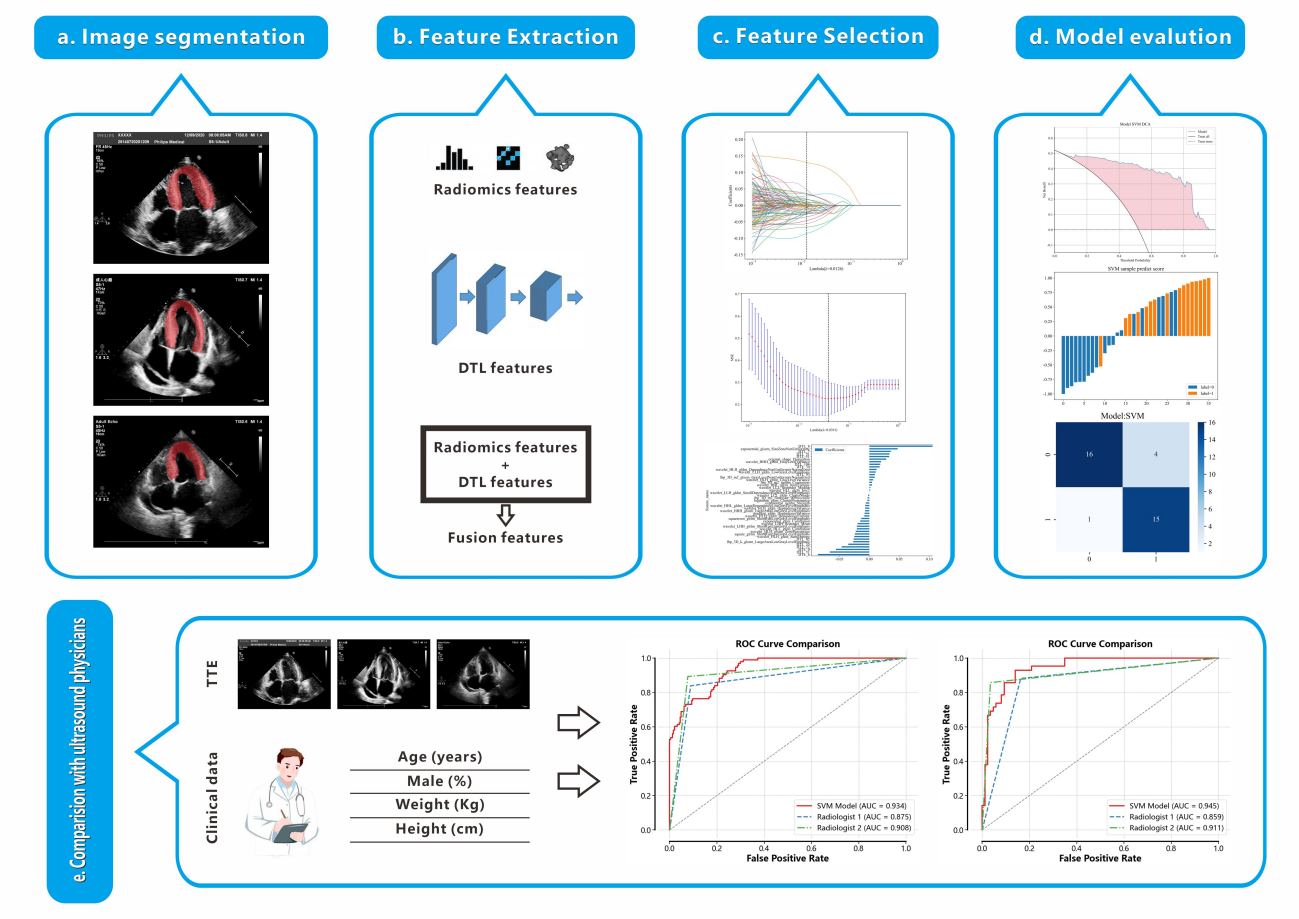
**Workflow of this study**. (a) Delineation of the left ventricular myocardium ROI. (b) Extraction of 1561 radiomics features and 128 DTL features from the ROI, followed by their fusion. (c) Feature selection using the LASSO method. (d) Construction of a model based on the selected features to differentiate between HCM and non-HCM. (e) Comparison of the performance of the RAD+DTL model with that of echocardiologists. TTE, transthoracic echocardiography; ROC, receiver operating characteristic; ROI, region of interest; DTL, deep transfer learning; HCM, hypertrophic cardiomyopathy; RAD, radiomics.

The flowchart of this study is shown in Fig. 1.

### 2.1 Patient Cohort

**Fig. 2.**
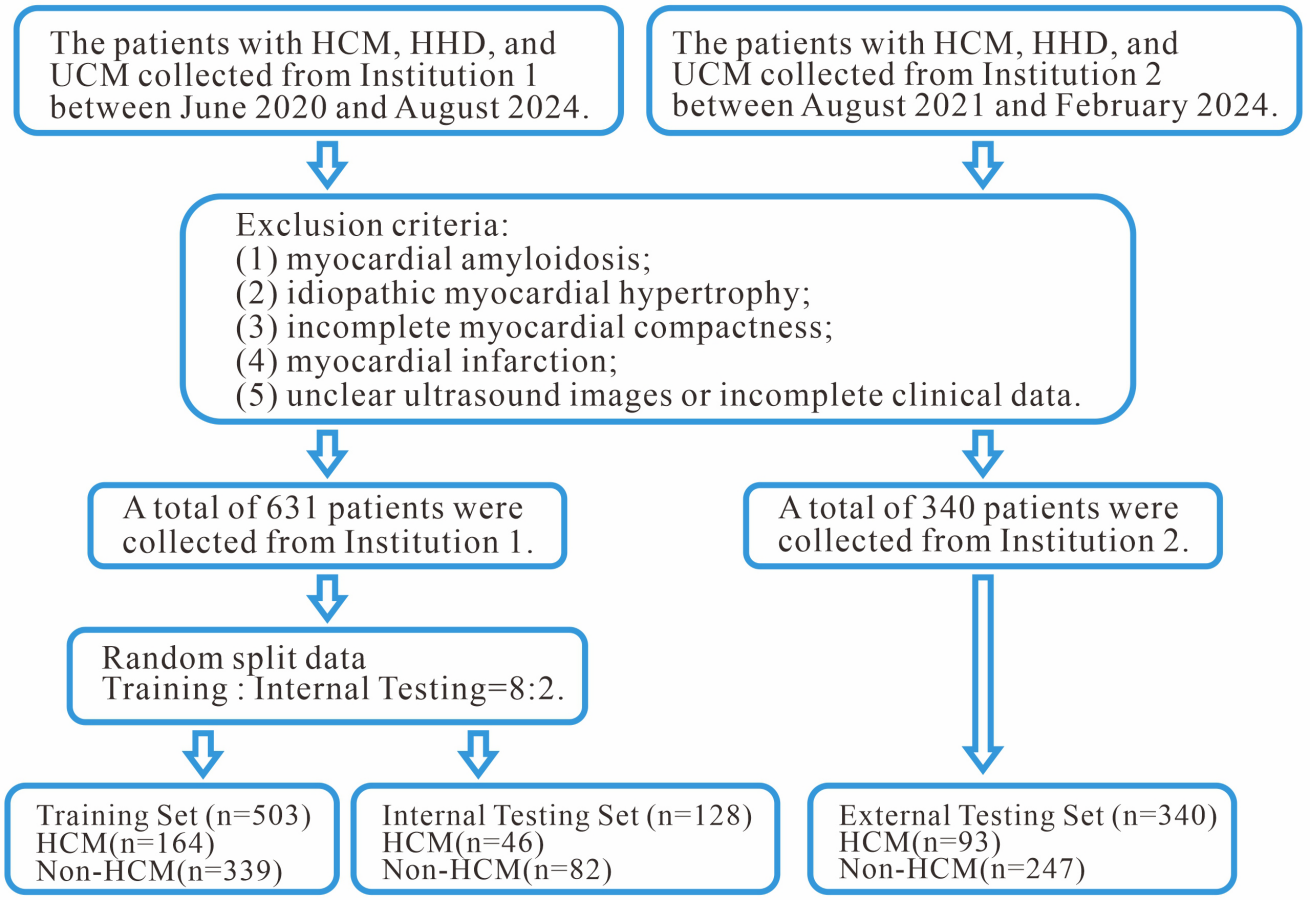
**The flowchart illustrates the participant selection process and exclusion criteria**. HHD, hypertensive heart disease; UCM, uremic cardiomyopathy.

This study is a retrospective study, and all relevant data were anonymized and extracted solely from the patients’ existing medical records. Since the study did not involve direct patient contact or additional medical procedures, the study was exempted by the Ethics Committee of the First Affiliated Hospital of Wannan Medical College, and patient informed consent was not required. The entire study strictly adhered to the relevant regulations of the Declaration of Helsinki (2013 revision). All participants were from Institution 1 and Institution 2, both of which participated in the study. The study period spanned from June 2020 to August 2024, during which 2036 patients’ TTE and clinical data were collected. A total of 971 patients were selected for the final analysis, with 631 patients from Institution 1 (HCM = 210, HHD = 216, UCM = 205) and 340 patients from Institution 2 (HCM = 93, HHD = 147, UCM = 100). Patients were divided into the HCM group and the non-HCM group based on the underlying cause. Patients from Institution 1 were randomly assigned to a training set (n = 503) and an internal validation set (n = 128) in an 8:2 ratio; all patients from Institution 2 formed the external validation set (n = 340). Inclusion criteria for HCM were: (1) end-diastolic left ventricular wall thickness (LVWT) ≥15 mm, not due to load conditions; (2) LVWT of 13–14 mm with a family history of HCM, not due to load conditions; (3) presence of pathogenic mutations in genetic Validation (e.g., MYH7 and MYBPC3). Inclusion criteria for HHD were: (1) LVWT >12 mm, with a confirmed diagnosis of hypertension; (2) patients diagnosed with HHD must not have other heart diseases that could lead to myocardial hypertrophy (e.g., valvular heart disease, aortic valve stenosis, athletic heart syndrome, and other types of cardiomyopathies). Inclusion criteria for UCM were a glomerular filtration rate (eGFR) <15 mL/min/1.73 m^2^. Exclusion criteria included: (1) myocardial amyloidosis; (2) unknown cause of myocardial hypertrophy; (3) myocardial disarray; (4) myocardial infarction; (5) unclear echocardiographic images or missing clinical data. The process of inclusion and exclusion of experimental subjects is shown in Fig. 2.

### 2.2 Echocardiogram Acquisition

This study followed the relevant guidelines published by the American Society of Echocardiography (ASE) and was conducted by two experienced echocardiologists (Echocardiologist 1 with 8 years of clinical experience and Echocardiologist 2 with 20 years of clinical experience) responsible for TTE image acquisition. Imaging was performed using Philips EPIQ 7C (Philips, Amsterdam, Netherlands) and GE Vivid E95 (GE, Boston, MA, USA) ultrasound machines, and the apical 4-chamber (A4C) view was selected for imaging. During the image acquisition process, the image quality was ensured to meet the required standards, and the relevant ultrasound parameters were accurately measured. The TTE parameters collected included left ventricular dimension at end-diastole (LVDd), left atrial dimension (LAD), left ventricular ejection fraction (LVEF), interventricular septal thickness (IVS), and left ventricular posterior wall thickness (LVPWT).

### 2.3 Image Preprocessing and Delineation of the Region of Interest

This study first standardizes the TTE images and then imports the processed A4C view images into the ITK-SNAP software (version 3.8, Philadelphia, PA, USA) for further analysis. Subsequently, Ultrasound Physician 1 uses software to delineate the left ventricular myocardium as the region of interest (ROI). Ultrasound Physician 2 reviews the ROI delineated by Ultrasound Physician 1, and in case of any discrepancies, the judgment of Ultrasound Physician 2 is considered final.

### 2.4 Radiomic Features Extraction

This study utilizes the open-source tool Pyradiomics package of Python Software (version 3.12, Python Software Foundation, DE, USA) for the extraction of radiomic features. A total of 1561 radiomic features were extracted through the analysis of the ROI regions, which can be categorized into three main types: geometric features, intensity features, and texture features. Geometric features describe the morphology of the delineated region, intensity features reflect the distribution of voxel intensities within the region, and texture features reveal the patterns of intensity or higher-order spatial distributions. The methods used for texture feature extraction include the Gray Level Co-occurrence Matrix (GLCM), Gray Level Run Length Matrix (GLRLM), Gray Level Size Zone Matrix (GLSZM), and Neighborhood Gray Tone Difference Matrix (NGTDM). These methods allow for the extraction of detailed texture information from the ROI regions, thereby contributing to more accurate regional descriptions and diagnoses. To evaluate the reproducibility of the radiomic feature extraction, two ultrasound physicians randomly selected ultrasound images from 50 patients, re-delineated the ROIs, and calculated the intra-class correlation coefficient (ICC). Features were considered to have high reproducibility when the ICC value was greater than 0.75.

### 2.5 Deep Transfer Learning Features Extraction

Before performing deep learning feature extraction, a minimal bounding rectangle that encompasses the ROI region must first be cropped. For model construction, this study selected DenseNet121 as the base network for transfer learning due to its outstanding performance on the ImageNet dataset. The DenseNet121 model was first loaded, retaining the weights of the initial convolutional layers, while freezing the parameters of these layers. Next, the final fully connected layer of the model was replaced with a new layer to ensure that its output matched the number of classes in the target dataset. To further enhance the model’s diagnostic performance, cross-entropy loss was used for fine-tuning. During the optimization process, the Adam optimizer was chosen, with a batch size of 64, a learning rate of 0.001, and 50 epochs of training. After fine-tuning, the parameters of the network were frozen to ensure the stability and consistency of the feature extraction process. Finally, by extracting the output from the second-to-last layer of the model, 50,177 DTL features were obtained for each patient.

### 2.6 Features Selection

**Fig. 3.**
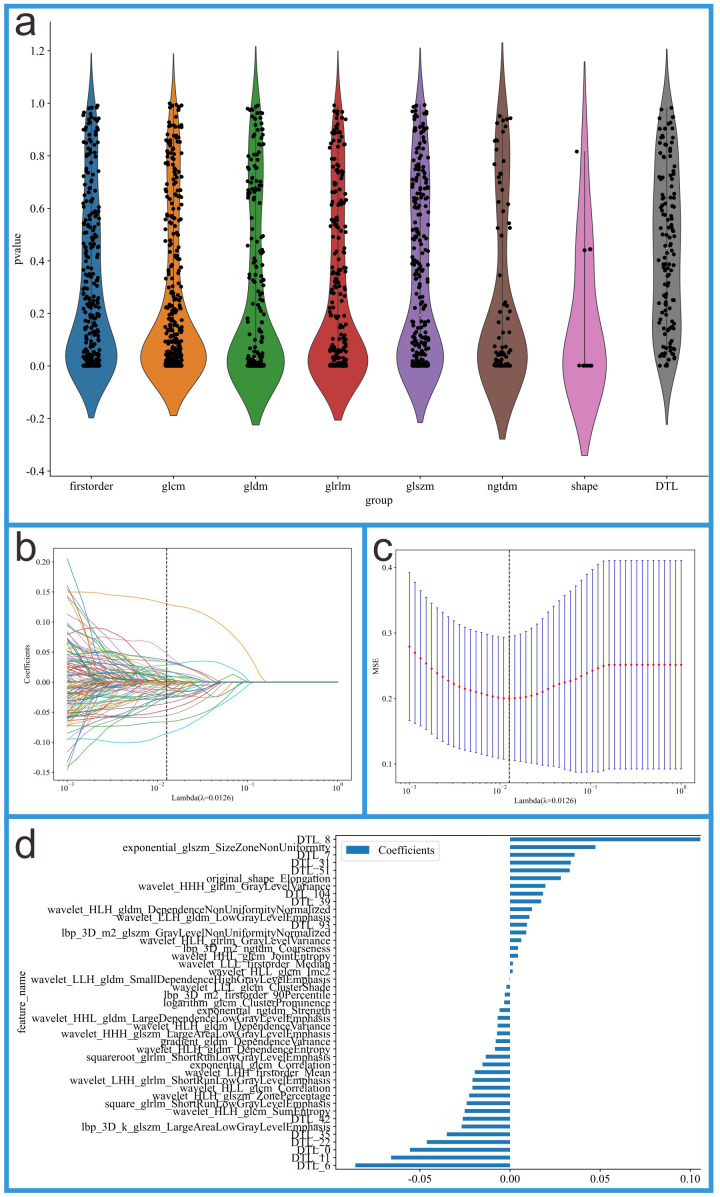
**Feature selection process for the integrated feature dataset**. (a) Feature distribution plot. (b) Coefficients from 10-fold cross-validation. (c) Mean squared error (MSE) from 10-fold cross-validation. (d) Feature weights of non-zero coefficients.

This study first performed dimensionality reduction on DTL features using Principal Component Analysis (PCA), ultimately retaining 128 features. Subsequently, the 128 DTL features were fused with 1561 radiomics features to create a combined feature dataset. Next, an independent samples *t*-test was conducted on all features, and significant features with *p*-values less than 0.05 were selected. Following this, a LASSO regression model was employed for feature selection, using the regularization parameter λ to shrink the regression coefficients to zero, thereby eliminating many irrelevant features. To determine the optimal λ value, 10-fold cross-validation was used to select the λ value that minimized the validation error. Ultimately, the features with non-zero regression coefficients were retained, and LASSO modeling was performed using the scikit-learn library in Python. To address high correlation between features, Spearman’s rank correlation coefficient was used to assess relationships between features, and features with correlation coefficients exceeding 0.9 with other features were removed. To further enhance the accuracy of feature selection, a greedy recursive elimination method was employed, which removed the most redundant features in each iteration. After feature selection, 30 radiomics features and 13 DTL features were obtained. The entire feature selection process and the final selected features are shown in Fig. 3.

### 2.7 Model Construction

**Fig. 4.**
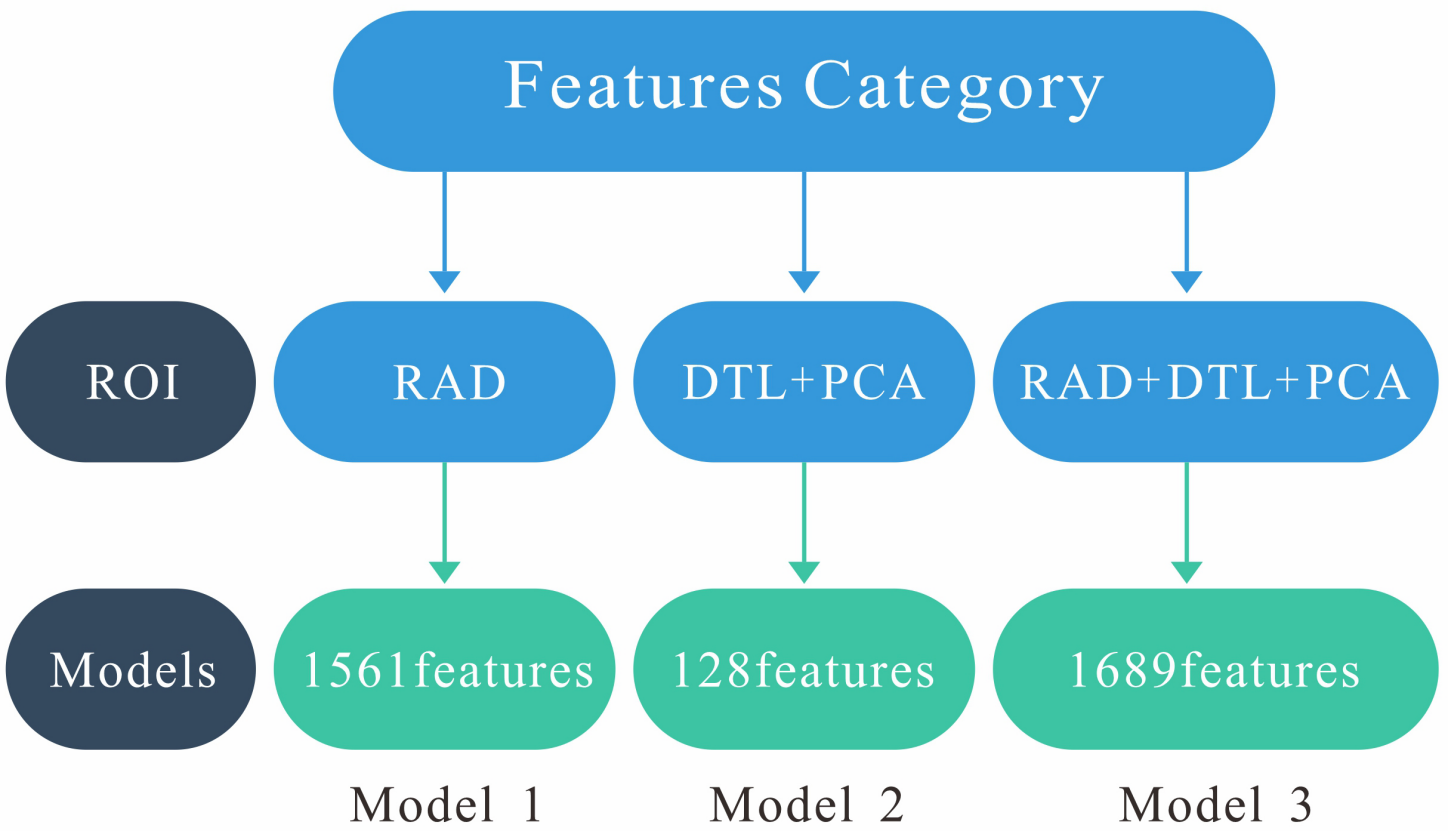
**Structural schematic of these three models**. Model 1 (RAD): 1561 radiomics features. Model 2 (DTL+PCA): 128 deep transfer learning features. Model 3 (RAD+DTL+PCA): 1561 radiomics features and 128 deep transfer learning features. PCA, principal component analysis.

Model 1 was constructed using radiomics features extracted from the ROI region. Model 2 was built based on DTL features extracted from the ROI region. Model 3 (fusion model) combines radiomics features and DTL features. Fig. 4 illustrates the structural schematic of these three models.

### 2.8 Model Comparison

This study employs multiple evaluation metrics to assess the performance of each model, including area under the curve (AUC), receiver operating characteristic (ROC) curve, accuracy, sensitivity, specificity, decision curve analysis (DCA), predictive scores, and 95% confidence intervals (95% CI). To compare the differences in ROC curves between different models, the DeLong test was applied in this study. To select the best machine learning algorithm, the radiomics features from Model 1 were input into ten machine learning algorithms, and the optimal algorithm was determined by comparing their performance. After selecting the best algorithm, the features from Models 2 and 3 were also modeled using the same algorithm. To validate the effectiveness of Model 3, two experienced ultrasound doctors were invited to perform the evaluation (Doctor 1 with 8 years of experience and Doctor 2 with 20 years of experience). During the evaluation process, both doctors classified the patients into the HCM or non-HCM group based on the TTE images, TTE parameters, and clinical information, without knowledge of the patient’s diagnosis. Finally, the diagnostic results of Model 3 were compared and analyzed with the diagnostic results of the two ultrasound doctors.

### 2.9 Statistical Analysis

This study performed data analysis using R software (version 4.0.2, R Foundation for Statistical Computing, Vienna, Austria) and Python. Continuous variables are expressed as mean ± standard deviation (SD). Feature selection was carried out using the χ^2^ test, and independent samples *t*-test, with a *p*-value less than 0.05 considered statistically significant. The reproducibility of the experiments was assessed using the ICC, and results were considered statistically significant only when the ICC value was greater than 0.75. ROC curves and AUC values were plotted and calculated using the scikit-learn package in Python to evaluate the diagnostic accuracy of the internal validation set and external validation set. DCA was employed to assess the clinical utility of the predictive model.

## 3. Results

### 3.1 Patient Characteristics and Clinical Features

This study collected clinical data and echocardiographic parameters from 971 patients, with detailed information provided in Table 1. The patients were divided into two groups: 303 cases in the HCM group and 668 cases in the non-HCM group. Independent samples *t*-test, Mann-Whitney U test, and chi-square test were used to compare the clinical characteristics between the two groups. The results indicated that there were significant differences in IVS thickness, age and LVEF (*p* < 0.05). However, no significant differences were found in LVDd, LAD, LVPWT, or gender (*p* > 0.05).

**Table 1.** **The patient’s clinical data and echocardiographic parameters**.

Variables	Training Set (n = 503)			Internal Validation Set (n = 128)			External Validation Set (n = 340)		
	HCM (n = 164)	Non-HCM (n = 339)	*p*-value	HCM (n = 46)	Non-HCM (n = 82)	*p*-value	HCM (n = 93)	Non-HCM (n = 247)	*p*-value
Age (years)	59.90 ± 16.41	63.37 ± 14.44	0.016	65.80 ± 13.77	62.44 ± 13.84	0.185	60.36 ± 9.58	56.96 ± 14.12	0.012
Male (%)	57 (34.76)	136 (40.12)	0.246	23 (50.00)	36 (43.90)	0.507	64 (68.82)	148 (59.92)	0.131
Weight (Kg)	73.11 ± 14.33	74.11 ± 15.61	0.476	69.84 ± 11.51	67.37 ± 13.54	0.279	71.64 ± 12.31	72.77 ± 14.62	0.474
Height (cm)	167.21 ± 9.08	166.57 ± 8.94	0.457	168.83 ± 8.59	169.33 ± 8.67	0.758	167.94 ± 8.26	168.96 ± 9.32	0.327
LVDd (mm)	50.62 ± 7.12	51.01 ± 7.92	0.580	49.70 ± 6.17	49.88 ± 5.71	0.871	50.21 ± 7.59	50.99 ± 8.03	0.406
LAD (mm)	42.51 ± 7.30	41.72 ± 8.48	0.282	44.95 ± 8.88	40.38 ± 6.28	0.003	43.76 ± 8.06	42.11 ± 7.25	0.085
LVEF (%)	67.35 ± 7.84	59.89 ± 8.19	<0.001	68.40 ± 6.56	62.19 ± 5.53	<0.001	69.16 ± 8.36	62.89 ± 9.21	<0.001
IVS (mm)	18.10 ± 2.28	14.07 ± 2.26	<0.001	18.85 ± 3.36	14.31 ± 1.92	<0.001	18.4 3 ± 3.64	13.62 ± 5.11	<0.001
LVPWT (mm)	13.49 ± 2.13	13.72 ± 2.04	0.251	12.85 ± 1.60	13.69 ± 1.78	0.007	14.19 ± 2.48	13.89 ± 1.97	0.296

The clinical characteristics include age, IVS thickness, LVEF, LVDd, LAD, LVPWT, and gender. The results showed that IVS thickness and LVEF were significantly different between the two groups (*p* < 0.05), while no significant differences were found in age, LVDd, LAD, LVPWT, or gender (*p* > 0.05). LVDd, left ventricular dimension at end-diastole; LAD, left atrial dimension; LVEF, left ventricular ejection fraction; IVS, interventricular septal thickness; LVPWT, left ventricular posterior wall thickness.

### 3.2 Machine Learning Model Selection

This study utilized ten machine learning algorithms, including logistic regression (LR), SVM, Naive Bayes, K-nearest neighbors (KNN), RandomForest, LightGBM, ExtraTrees, Gradient Boosting, XGBoost, and AdaBoost, which were evaluated as shown in Table 2. Due to overfitting issues, RandomForest, ExtraTrees, and XGBoost were excluded. In the training set of Model 1, LightGBM achieved the highest AUC of 0.944, outperforming Gradient Boosting (AUC = 0.895), KNN (AUC = 0.849), and SVM (AUC = 0.836). In the internal validation set, SVM achieved the highest AUC of 0.853. In the external validation set, SVM again achieved the highest AUC of 0.772. Based on these results, to enhance the stability and reliability of the model, SVM was ultimately selected as the optimal machine learning algorithm for building Model 1. SVM was also chosen as the algorithm for constructing Models 2 and 3.

**Table 2.** **Evaluation of the performance of ten machine learning models constructed using radiomics features**.

Model name	Task	AUC (95% CI)	Accuracy	Sensitivity	Specificity	F1	PPV	NPV
LR	Training Set	0.749 (0.705–0.792)	0.704	0.699	0.706	0.689	0.745	0.739
LR	Internal Validation Set	0.787 (0.705–0.870)	0.750	0.667	0.791	0.864	0.681	0.763
LR	External Validation Set	0.767 (0.710–0.824)	0.685	0.774	0.652	0.520	0.394	0.906
NaiveBayes	Training Set	0.722 (0.676–0.769)	0.698	0.633	0.730	0.691	0.378	0.948
NaiveBayes	Internal Validation Set	0.752 (0.662–0.843)	0.688	0.762	0.651	0.795	0.554	0.827
NaiveBayes	External Validation Set	0.702 (0.641–0.762)	0.709	0.591	0.753	0.725	0.469	0.813
SVM	Training Set	0.836 (0.798–0.873)	0.751	0.849	0.703	0.815	0.786	0.797
SVM	Internal Validation Set	0.853 (0.786–0.920)	0.750	0.833	0.709	0.882	0.802	0.821
SVM	External Validation Set	0.772 (0.714–0.831)	0.729	0.710	0.737	0.816	0.681	0.763
KNN	Training Set	0.849 (0.817–0.882)	0.791	0.627	0.872	0.817	0.786	0.797
KNN	Internal Validation Set	0.749 (0.663–0.835)	0.703	0.524	0.791	0.792	0.739	0.733
KNN	External Validation Set	0.716 (0.659–0.773)	0.706	0.538	0.769	0.689	0.533	0.852
RandomForest	Training Set	0.998 (0.996–0.999)	0.976	0.970	0.979	0.957	0.973	0.991
RandomForest	Internal Validation Set	0.752 (0.661–0.844)	0.688	0.571	0.744	0.783	0.387	0.717
RandomForest	External Validation Set	0.664 (0.599–0.730)	0.685	0.387	0.798	0.761	0.561	0.682
ExtraTrees	Training Set	1.000 (1.000–1.000)	0.670	0.000	1.000	0.962	0.989	0.976
ExtraTrees	Internal Validation Set	0.794 (0.713–0.875)	0.711	0.524	0.802	0.750	0.543	0.849
ExtraTrees	External Validation Set	0.671 (0.611–0.731)	0.647	0.581	0.672	0.715	0.669	0.673
XGBoost	Training Set	1.000 (0.999–1.000)	0.988	0.994	0.985	0.966	0.969	0.985
XGBoost	Internal Validation Set	0.767 (0.680–0.854)	0.719	0.690	0.733	0.799	0.399	0.723
XGBoost	External Validation Set	0.720 (0.661–0.779)	0.682	0.667	0.688	0.691	0.332	0.714
LightGBM	Training Set	0.944 (0.925–0.964)	0.889	0.855	0.905	0.904	0.930	0.774
LightGBM	Internal Validation Set	0.791 (0.709–0.872)	0.680	0.810	0.616	0.866	0.951	0.618
LightGBM	External Validation Set	0.705 (0.645–0.765)	0.632	0.742	0.591	0.736	0.204	0.953
GradientBoosting	Training Set	0.895 (0.866–0.923)	0.809	0.831	0.798	0.892	0.892	0.749
GradientBoosting	Internal Validation Set	0.788 (0.706–0.871)	0.734	0.619	0.791	0.812	0.831	0.657
GradientBoosting	External Validation Set	0.686 (0.624–0.748)	0.697	0.538	0.757	0.769	0.801	0.587
AdaBoost	Training Set	0.803 (0.765–0.841)	0.728	0.783	0.700	0.782	0.752	0.759
AdaBoost	Internal Validation Set	0.776 (0.682–0.869)	0.781	0.714	0.814	0.755	0.841	0.662
AdaBoost	External Validation Set	0.678 (0.619–0.738)	0.562	0.796	0.474	0.632	0.910	0.705

The performance metrics for evaluating the machine learning model include AUC (95% CI), accuracy, sensitivity, specificity, F1 score, PPV and NPV. LR, logistic regression; PPV, positive predictive value; NPV, negative predictive value; KNN, K-nearest neighbors.

### 3.3 Model Performance Evaluation

**Fig. 5.**
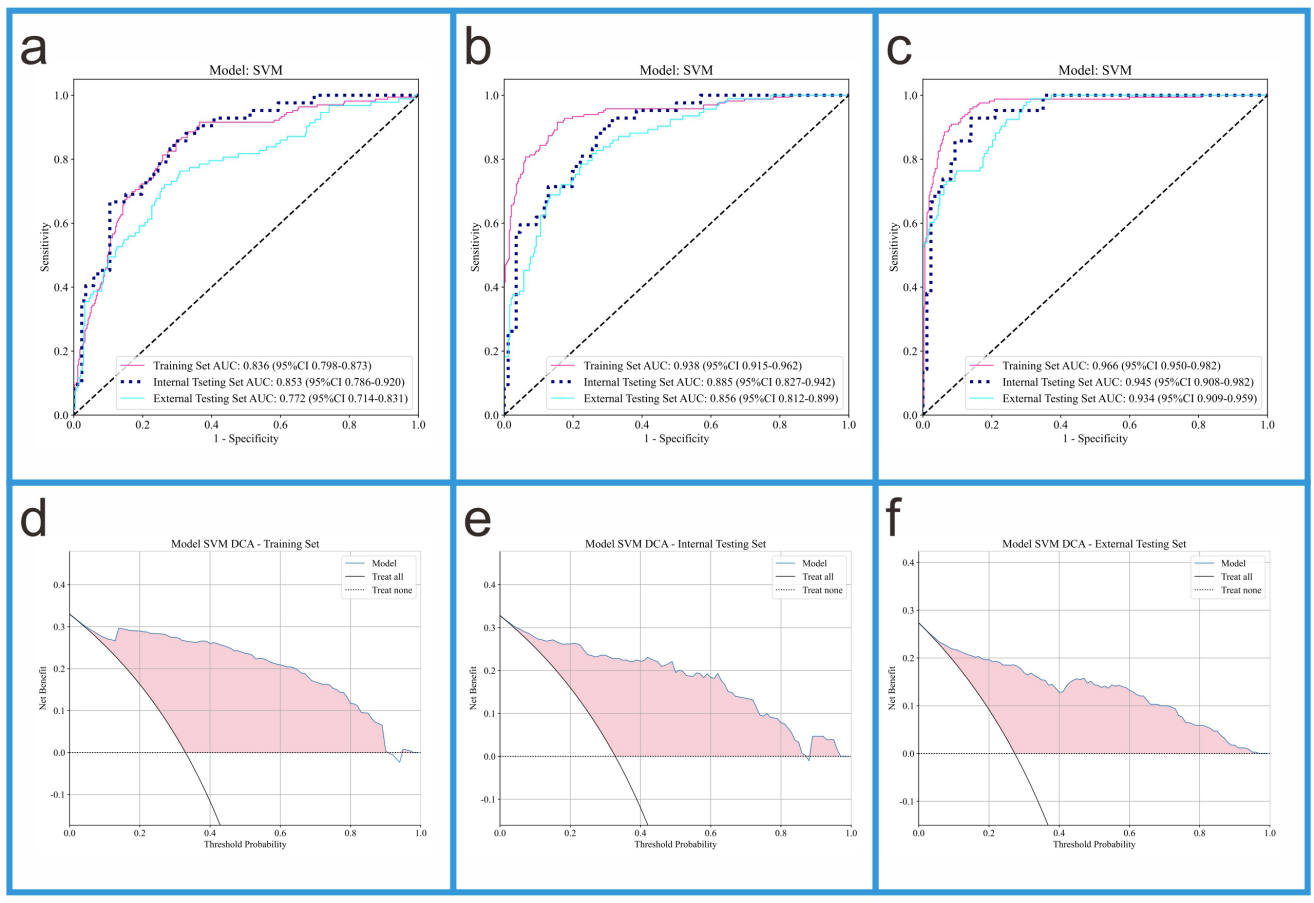
**ROC curves of three models and DCA curves of Model 3**. (a) The ROC curves of Model 1. (b) The ROC curves of Model 2. (c) The ROC curves of Model 3. (d) The DCA curve of Model 3 in the training set. (e) The DCA curve of Model 3 in the internal validation set. (f) The DCA curve of Model 3 in the external validation set.

This study evaluated the performance of Models 1, 2, and 3 using AUC (95% CI), accuracy, sensitivity, and specificity (see Table 3). In the training set, the AUC values for Models 1, 2, and 3 were 0.836, 0.938, and 0.966, respectively. In the internal validation set, the AUC values for Models 1, 2, and 3 were 0.853, 0.885, and 0.945, respectively. In the external validation set, the AUC values for Models 1, 2, and 3 were 0.772, 0.856, and 0.934, respectively. In the training set, Model 3 (AUC = 0.966) performed the best, outperforming Model 1 (AUC = 0.938) and Model 2 (AUC = 0.836); in the internal validation set, Model 3 (AUC = 0.945) performed the best, outperforming Model 1 (AUC = 0.885) and Model 2 (AUC = 0.853); in the external validation set, Model 3 (AUC = 0.934) also outperformed Model 1 (AUC = 0.856) and Model 2 (AUC = 0.772). These results suggest that Model 3, which integrates radiomic features and DTL features, significantly outperforms Models 1 and 2, which rely solely on radiomic features and DTL features, respectively. The ROC curves for Models 1, 2, and 3, along with the DCA curve for Model 3, are shown in Fig. 5.

**Table 3.** **Evaluation of the performance of the Model 1, Model 2, Model 3**.

Models	Task	AUC (95% CI)	Accuracy	Sensitivity	Specificity	F1	PPV	NPV
Model 1 (RAD)	Training Set	0.836 (0.798–0.873)	0.751	0.849	0.703	0.815	0.786	0.797
Model 1 (RAD)	Internal Validation Set	0.853 (0.786–0.920)	0.750	0.833	0.709	0.882	0.802	0.821
Model 1 (RAD)	External Validation Set	0.772 (0.714–0.831)	0.729	0.710	0.737	0.816	0.681	0.763
Model 2 (DTL)	Training Set	0.938 (0.915–0.962)	0.867	0.910	0.846	0.889	0.850	0.830
Model 2 (DTL)	Internal Validation Set	0.885 (0.827–0.942)	0.758	0.905	0.686	0.824	0.795	0.803
Model 2 (DTL)	External Validation Set	0.856 (0.812–0.899)	0.776	0.774	0.777	0.888	0.789	0.799
Model 3 (RAD+DTL)	Training Set	0.966 (0.950–0.982)	0.913	0.904	0.917	0.922	0.860	0.953
Model 3 (RAD+DTL)	Internal Validation Set	0.945 (0.908–0.982)	0.875	0.905	0.860	0.904	0.941	0.872
Model 3 (RAD+DTL)	External Validation Set	0.934 (0.909–0.959)	0.800	0.914	0.757	0.913	0.833	0.826

The performance metrics for evaluating the RAD model, DTL model and RAD+DTL models include AUC (95% CI), accuracy, sensitivity, specificity, F1 score, PPV and NPV.

**Fig. 6.**
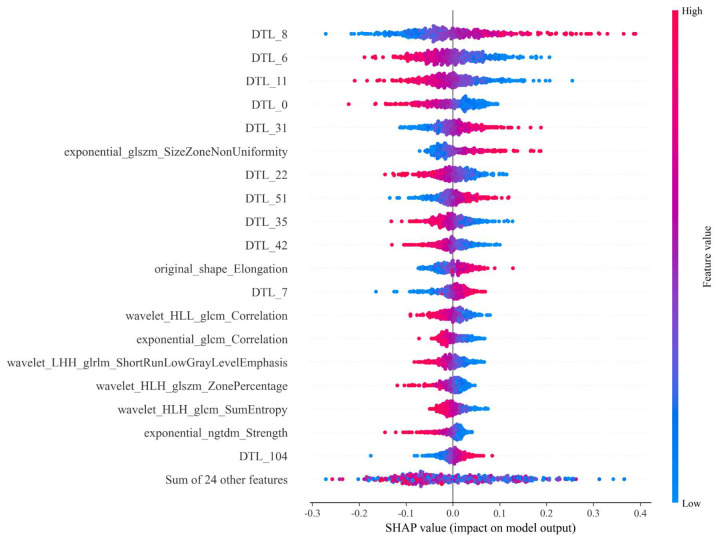
**The SHAP analysis plot of Model 3**.

We conducted SHAP analysis on Models3 to enhance the interpretability of the models. Fig. 6 presents the SHAP analysis results for Model 3.

### 3.4 Comparison With Ultrasound Physicians

**Fig. 7.**
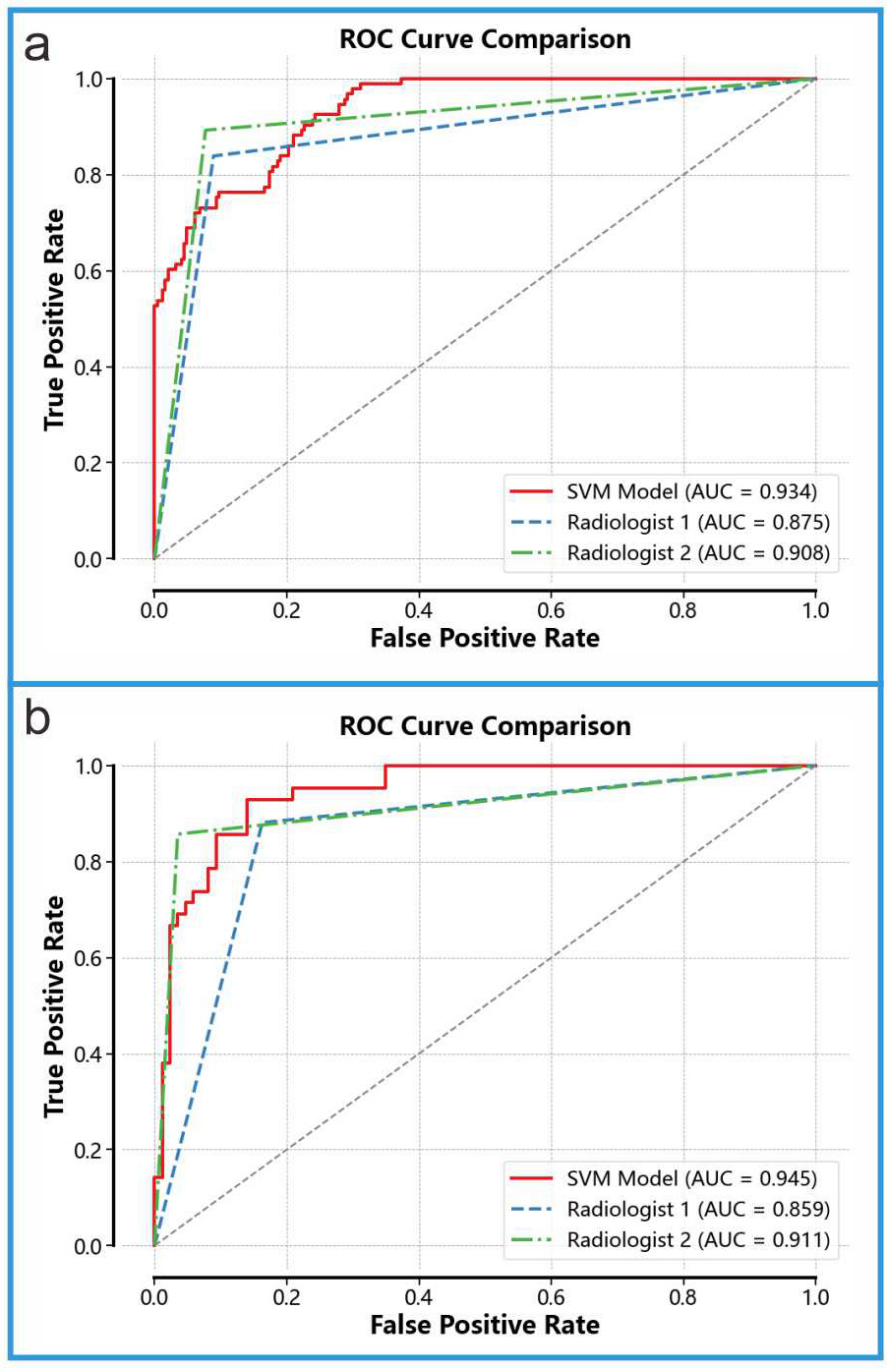
**Comparison With Ultrasound Physicians**. (a) Diagnostic performance of Model 3 and the two ultrasound physicians on the internal validation set. (b) Diagnostic performance of Model 3 and the two ultrasound physicians on the external validation set.

We compared the diagnostic performance of Model 3 with that of two ultrasound physicians (see Fig. 7). On the internal validation set, Model 3 achieved an AUC of 0.945, significantly outperforming Ultrasound Physician 1 (AUC = 0.859) and Ultrasound Physician 2 (AUC = 0.911). On the external validation set, Model 3 achieved an AUC of 0.934, also surpassing Ultrasound Physician 1 (AUC = 0.875) and Ultrasound Physician 2 (AUC = 0.908). To further compare the diagnostic capabilities of Model 3 and the ultrasound physicians, we performed the Delong test (results shown in Table 4). The DeLong test can help determine whether there is a significant difference between the diagnostic performance of the fusion model and that of ultrasound physicians when comparing the two. In this study, the results of the DeLong test indicated that the fusion model significantly outperformed the diagnostic performance of ultrasound physicians.

**Table 4.** **The Delong test results for Model 3 and the ultrasound physicians**.

Model	Task	Ultrasound Physicians	*p*-value
Model 3 (RAD+DTL)	Internal Validation Set	Ultrasound Physician 1	<0.001
		Ultrasound Physician 2	<0.001
	External Validation Set	Ultrasound Physician 1	<0.001
		Ultrasound Physician 2	<0.001

The results of the DeLong test indicated that the fusion model significantly outperformed the diagnostic performance of ultrasound physicians.

## 4. Discussion

This study developed an efficient diagnostic model based on the A4C images from 971 patients, incorporating radiomic features and DTL features. The model demonstrated excellent performance in distinguishing HCM from LVH caused by other factors, outperforming two experienced ultrasound physicians in diagnostic ability. These results suggest that a diagnostic model developed using TTE, combined with radiomic and DTL features, can effectively differentiate HCM from other causes of LVH, thereby improving diagnostic efficiency.

HCM and other causes of LVH, such as HHD and UCM, differ significantly in terms of etiology, treatment, and prognosis. HCM is typically caused by sarcomere mutations and myofibrillar disarray, while HHD is primarily induced by chronic hypertension, leading to increased afterload on the left ventricle and subsequent LVH [1, 5]. UCM, on the other hand, is caused by uremia [4]. The treatment of HCM focuses on assessing the risk of sudden cardiac death and implementing primary or secondary preventive measures, while addressing complications such as arrhythmias, heart failure, or left ventricular outflow tract obstruction, along with family screening and counseling [1, 31]. In contrast, the treatment for HHD emphasizes blood pressure control, alleviating cardiac burden, and preventing related complications [5]. The primary treatment for UCM is dialysis or kidney transplantation [4]. Despite the significant differences in etiology, treatment, and prognosis, HCM and LVH due to other causes often present similarly on TTE, complicating diagnosis when relying solely on this imaging modality. The diagnostic challenge is further heightened by the high prevalence of hypertension in both HCM and UCM patients, often requiring invasive or costly diagnostic procedures [32, 33, 34]. Therefore, developing a novel diagnostic model based on TTE to differentiate HCM from other causes of LVH is of great significance. This approach would not only facilitate rapid and accurate diagnosis but also enable timely, targeted treatment for patients, thereby reducing healthcare costs.

We developed a diagnostic model based on TTE to extract radiomics features and DTL features, which demonstrated excellent performance in distinguishing HCM from other causes of LVH. The model’s performance across different datasets is as follows: the training set achieved an AUC of 0.966 and an accuracy of 0.913; the internal validation set achieved an AUC of 0.945 and an accuracy of 0.875; and the external validation set achieved an AUC of 0.934 and an accuracy of 0.8. Our model’s AUC in the training set (0.966) is higher than that in the external validation set (0.934). This suggests a potential overfitting issue. In future studies, we will incorporate more external validation sets to further assess our model’s performance. In comparison, the model proposed by Wang *et al*. [26] based on ResNet, utilizes MRI T1 images to extract deep learning features to differentiate HCM and HHD, achieving an AUC of 0.83. Compared to this model, our model not only has advantages in diagnostic accuracy and the range of applicable diseases, but also significantly reduces the economic burden on patients, as TTE is more cost-effective than MRI. Additionally, Xu *et al*. [29] developed a deep learning algorithm based on ResUNet to identify the causes of LVH using TTE video images, with an AUC of 0.869. Our approach, however, demonstrates stronger diagnostic capability, and since we extract features from static TTE images, it is easier to standardize, requires lower computational demand, and is more practical than extracting features from video images. Bao *et al*. [35] developed a novel nomogram based on echocardiography for the simplified classification of cardiac tumors, which integrates radiomics features and clinical characteristics. Our study did not incorporate clinical features; however, we included DTL features, and our research is a multicenter study, which enhances the credibility of our research [35]. Previous studies have mostly focused on extracting a single type of radiomics or deep learning feature, whereas we have constructed a more comprehensive feature set by combining radiomics features with DTL features. This approach not only allows us to leverage the low-level, structured information obtained from radiomics but also integrates the high-level information from DTL models. Our experimental results indicate that the fusion of radiomics features and DTL features effectively enhances the strengths of each, significantly improving the diagnostic performance of the model. This strategy of feature fusion has also achieved significant success in other related studies [9, 22, 23]. Our model does not require additional tests and only needs the most common four-chamber view from echocardiography, making it easy to operate. In many economically underdeveloped regions, our model is more applicable, cost-effective, and easier to promote.

This study has several limitations. First, only HHD and UCM were considered as diseases for differentiating HCM, excluding normal subjects and other rare causes of LVH, such as myocardial amyloidosis, incomplete left ventricular compactness, Fabry disease, and Danon syndrome. The exclusion of these normal subjects and other causes of LVH may affect the general applicability of our model, as the causes of LVH diagnosed in hospitals are unknown, and our model is only capable of identifying specific causes of LVH. In the future, we plan to expand the scope of the study to include more cases of LVH from different etiologies and normal subjects to enhance the generalizability and persuasiveness of the research. Secondly, manual segmentation was used in the study to extract features. Although we employ double review to minimize errors, there is still a certain degree of subjectivity. If we could achieve accurate automatic segmentation, it would greatly alleviate this issue. However, due to our current limitations, we have not yet mastered precise automatic segmentation technology. In the future, we will focus on developing more intelligent and precise automatic segmentation techniques to improve work efficiency, reduce human error, and enhance accuracy and consistency.

## 5. Conclusions

In conclusion, this study successfully developed an efficient diagnostic model by integrating radiomic features extracted from TTE with DTL features. The model demonstrated significant diagnostic ability in distinguishing HCM from LVH caused by other etiologies. Compared to experienced echocardiographers, the integrated model showed a distinct advantage in diagnostic performance, exhibiting higher AUC values and accuracy. Moreover, the performance of the integrated model outperformed that of single models relying solely on either radiomic features or DTL features. In conclusion, this study provides an innovative diagnostic tool for distinguishing HCM from LVH of different etiologies, offering more precise and efficient support for clinicians.

## Availability of Data and Materials

Due to the requirement to protect patient privacy, the datasets analyzed during this study are not publicly available. In accordance with relevant regulations, the data can be obtained from the corresponding author. For further data access, interested parties may contact through official channels, and data access will be provided in compliance with privacy protection policies.
